# α-Ketoglutarate supplementation and NAD+ modulation enhance metabolic rewiring and radiosensitization in SLC25A1 inhibited cancer cells

**DOI:** 10.1038/s41420-024-01805-x

**Published:** 2024-01-15

**Authors:** Kexu Xiang, Mikhail Kunin, Safa Larafa, Maike Busch, Nicole Dünker, Verena Jendrossek, Johann Matschke

**Affiliations:** 1 Institute of Cell Biology (Cancer Research), University Hospital Essen, University of Duisburg-Essen, 45147 Essen, Germany; 2 Department of Gastroenterology, Chongqing University Cancer Hospital, 400030 Chongqing, China; 3 Chongqing Key Laboratory of Translational Research for Cancer Metastasis and Individualized Treatment, Chongqing University Cancer Hospital, 400030 Chongqing, China; 4 Center for Translational Neuro- and Behavioral Sciences, Institute of Anatomy II, Department of Neuroanatomy, Medical Faculty, University of Duisburg-Essen, 45147 Essen, Germany; 5 German Cancer Consortium (DKTK) partner site Essen a partnership between DKFZ and University Hospital, Essen, Germany

**Keywords:** Cancer metabolism, Non-small-cell lung cancer

## Abstract

Metabolic rewiring is the result of the increasing demands and proliferation of cancer cells, leading to changes in the biological activities and responses to treatment of cancer cells. The mitochondrial citrate transport protein SLC25A1 is involved in metabolic reprogramming offering a strategy to induce metabolic bottlenecks relevant to radiosensitization through the accumulation of the oncometabolite D-2-hydroxyglutarate (D-2HG) upon SLC25A1 inhibition (SLC25A1i). Previous studies have revealed the comparative effects of SLC25A1i or cell-permeable D-2HG (octyl-D-2HG) treatments on DNA damage induction and repair, as well as on energy metabolism and cellular function, which are crucial for the long-term survival of irradiated cells. Here, α-ketoglutarate (αKG), the precursor of D-2HG, potentiated the effects observed upon SLC25A1i on DNA damage repair, cell function and long-term survival in vitro and in vivo, rendering NCI-H460 cancer cells more vulnerable to ionizing radiation. However, αKG treatment alone had little effect on these phenotypes. In addition, supplementation with nicotinamide (NAM), a precursor of NAD (including NAD^+^ and NADH), counteracted the effects of SLC25A1i or the combination of SLC25A1i with αKG, highlighting a potential importance of the NAD^+^/NADH balance on cellular activities relevant to the survival of irradiated cancer cells upon SLC25A1i. Furthermore, inhibition of histone lysine demethylases (KDMs), as a major factor affected upon SLC25A1i, by JIB04 treatment alone or in combination with αKG supplementation phenocopied the broad effects on mitochondrial and cellular function induced by SLC25A1i. Taken together, αKG supplementation potentiated the effects on cellular processes observed upon SLC25A1i and increased the cellular demand for NAD to rebalance the cellular state and ensure survival after irradiation. Future studies will elucidate the underlying metabolic reprogramming induced by SLC25A1i and provide novel therapeutic strategies for cancer treatment.

## Introduction

As an indispensable part of the biological activity of an organism, cellular metabolism is composed of the interaction of a series of metabolites to meet the needs of growth and homeostasis. In malignant cells, cellular metabolism undergoes metabolic reprogramming to adapt to the increasing demands and changes of redox homeostasis, that are critical for cell proliferation, metastasis and survival under applied treatment regimens [1–4]. During and after the transformation to a cancerous state, cells tend to rewire cellular metabolism to meet the increasing demands of cell growth and proliferation [5]. Due to the diversity of possible metabolic changes and the multiple interconnections of metabolic pathways of cancer cells, it appears to be challenging to describe an accurate static model of altered tumor metabolism that predicts the overall state of metabolic changes that support cancer cell growth [6]. Therefore, focusing on key metabolic processes may be a strategy to define cancer cell-dependent metabolic needs as well as treatment induced metabolic phenotypes [4]. In this context, mitochondria are important organelles that supply cells with energy and building blocks, but also regulate cellular activity by altering redox homeostasis and oncogenic signaling [7]. Furthermore, abnormal production of metabolites present in cancer cells but not in normal cells has been described to contribute to cancer initiation and progression [8]. It has been well established that the aberrant production of 2-hydroxyglutarate (2-HG), succinate and fumarate can induce cancer initiation and progression, linking these oncometabolites to cellular metabolic reprogramming and perturbation of biological processes [8–10]. However, the accumulation of oncometabolites has been linked to mutations in the respective producing enzymes (e.g. fumarate hydrotase (FH), succinate dehydrogenase (SDH) or isocitrate dehydrogenase (IDH)) [8]. Interestingly, our previous work revealed a strategy to induce 2-HG accumulation as a common phenotype by inhibiting the mitochondrial citrate carrier SLC25A1 in cancer cells without somatic mutation of IDH [11, 12]. SLC25A1 inhibition (SLC25A1i) created a phenotype represented by reduced repair of radiation-induced DNA double-strand breaks (DSBs) and reduced survival after radiotherapy (RT) [12]. More specifically, SLC25A1i impaired the repair of lethal DNA lesions introduced by ionizing radiation (IR) treatment, presumably by inducing the accumulation of the oncometabolite D-2-hydroxyglutarate (D-2HG) and the associated restriction of homologous recombination (HR) repair [12]. In addition, targeting SLC25A1 revealed a susceptibility of cancer cells to inhibition of poly(ADP-ribose)-polymerase (PARP)1 or the catalytic subunit of DNA-dependent protein kinase (DNA-PKcs) in combination with IR, suggesting a window of therapeutic opportunity [12]. Interestingly, SLC25A1i not only affected DNA repair, but also reduced the abundance of cellular NAD levels and mitochondrial function [12].

To interfere with the metabolic alterations induced by SLC25A1i, we used α-ketoglutarate (αKG) and nicotinamide (NAM) supplementation as a strategy to reverse the phenotype observed upon SLC25A1i and tested their ability to affect the biological activities of cancer cells alone or in combination with irradiation. In addition, we used JIB04 as a pan-inhibitor of histone lysine demethylases (KDMs) to recapitulate part of the observed phenotype induced by SLC25A1i.

## Results

### α-ketoglutarate (αKG) supplementation potentiated DNA damage and tumor growth delay of CTPI2-treated NCI-H460 cells

**Fig. 1:**
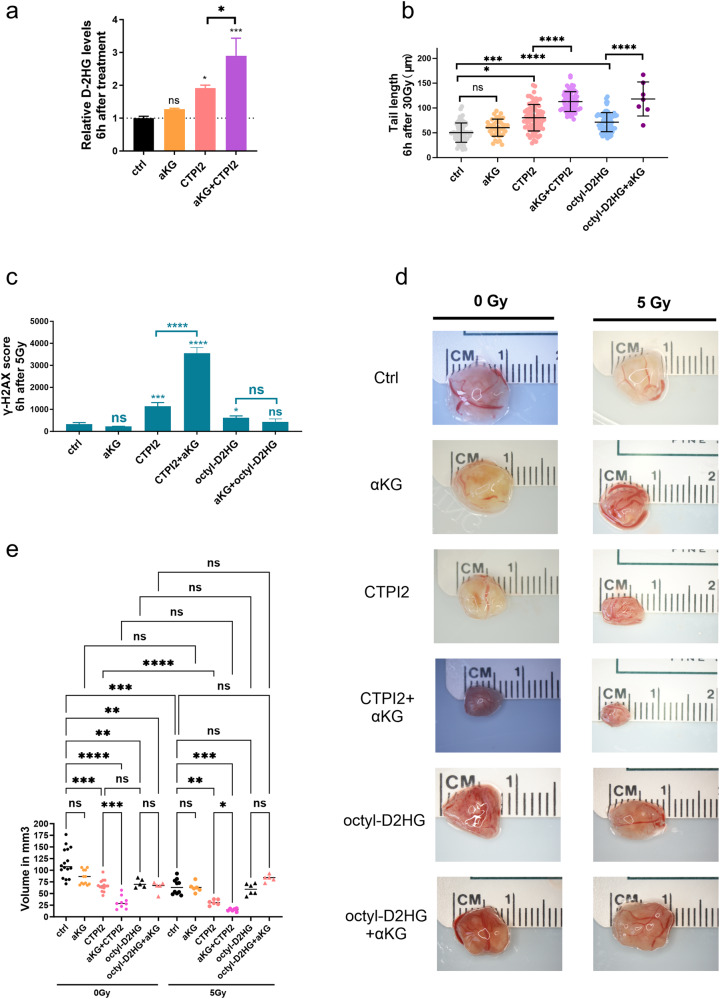
α-ketoglutarate (αKG) potentiates the phenotype induced by CTPI2 treatment. NCI-H460 cells were non-treated (ctrl) or pre-treated for 2 h with CTPI2 (200 μM) or octyl-D-2HG (150 μM), without or with αKG (8 mM) supplementation alone or in combination with ionizing radiation (IR) as indicated. **a** D-2HG production 6 h after CTPI2, αKG or the combination of CTPI2 and αKG treatment in NCI-H460 cell line, measured by the D-2HG assay kit. **b** Tail length in µm representing DNA damage induced by CTPI2, αKG, octyl-D-2HG treatment alone or in combination with αKG supplementation upon IR (30 Gy) as indicated. DNA damage was determined by alkaline comet assay 6 h after respective treatments in NCI-H460 cell line. **c** γ-H2AX score was measured by flow cytometry 6 h after CTPI2, αKG, octyl-D-2HG treatments alone or in combination with αKG supplementation upon IR (5 Gy) in NCI-H460. **d** Exemplary photomicrographs of NCI-H460 tumors dissected from CAM model 7 days after grafting representing the indicated treatments. **e** Quantification of tumor volumes acquired in the respective treatment groups. Data represent the mean values (±SD) from three independent experiments (*N* = 3). Statistical significance: by non-parametric unpaired t-test. ns=not significant (*p* > 0.05), *p < 0.05, **p < 0.01, ***p < 0.001, ****p < 0.0001. Asterisks above bars indicate comparison with respective control and parentheses above bars indicate significance between compared groups.

Since D-2HG acts as a competitive inhibitor of α-ketoglutarate-dependent dioxygenases (αKGDD) by replacing αKG as a substrate, we hypothesized that αKG supplementation after SLC25A1i or octyl-D-2HG treatment could reverse or rescue the observed effects on DNA damage response and alterations in cell function induced by the respective treatments. In our previous study, we have found that the 3rd generation small molecule inhibitor of SLC25A1, CTPI2, induced D-2HG accumulation and thereby impaired homologous recombination repair (HRR) [12]. In this study, we first supplemented αKG to the CTPI2-treated NCI-H460 lung cancer cell line in an attempt to modulate the D-2HG production. Here, αKG supplementation alone had no significant effect on the D-2HG production of the NCI-H460 cell line (Fig. 1a). Surprisingly, D-2HG production induced by CTPI2 treatment was significantly enhanced by additional αKG supplementation (Fig. 1a). Consistent with the increased accumulation of D-2HG, additional αKG supplementation in combination with CTPI2 treatment significantly potentiated the induction of radiation-induced DNA damage 6 h after irradiation as determined by the alkaline comet assay (Fig. 1b). Again, αKG supplementation alone had no significant effect on the radiation-induced DNA damage (Fig. 1b). To test, whether the observed induction of DNA damage by αKG supplementation was a consequence of D-2HG accumulation, we applied cell-permeable octyl-D-2HG treatment alone, as previously described [12], and in combination with additional αKG supplementation. Here, αKG supplementation potentiated radiation-induced DNA damage in the NCI-H460 cell line upon octyl-D-2HG treatment (Fig. 1b), suggesting D-2HG-related mechanisms that are potentiated by αKG treatment.

Next, we compared the ability of NCI-H460 cells to repair radiation-indued DSBs following CTPI2 or octyl-D-2HG treatment alone or in combination with αKG supplementation by quantifying radiation-induced γ-H2AX foci using flow cytometry and immunofluorescence as previously described [12]. Here, αKG supplementation in combination with CTPI2-treatment further enhanced the γ-H2AX signal induced by CTPI2 treatment alone at the 6 h post-irradiation timepoint at a dose of 5 Gy (Fig. 1c, Fig. S1g). Nevertheless, the increased level of radiation-induced γ-H2AX signal induced by octyl-D-2HG treatment at 6 h time point after irradiation with a dose of 5 Gy was not increased by additional αKG-supplementation, suggesting a more complex metabolic reprogramming induced by CTPI2 treatment compared to octyl-D-2HG treatment (Fig. 1c, Fig. S1a, b). The delay in the repair of radiation-induced γ-H2AX foci following CTPI2 or octyl-D-2HG treatment alone or in combination with αKG supplementation was confirmed in A549 lung cancer cell line, giving comparable results (Fig. S3a). Next, we used the CAM model as a proof-of-concept platform to validate the αKG-induced phenotype potentiation observed upon CTPI2 inhibition in vivo.

To investigate the ability of αKG supplementation to enhance the reduction in tumor growth of NCI-H460 cancer cells induced by CTPI2 treatment in vivo, we used the well-described chick embryo chorioallantoic membrane (CAM) model as previously reported [12–15]. Here, additional αKG supplementation further reduced the tumor volume of CTPI2-treated NCI-H460 cells (Fig. 1d, e). Notably, the additional application of IR further potentiated the reduction of tumor volume in NCI-H460 cells treated with both, CTPI2 and αKG (Fig. 1e). Interestingly, in the case of octyl-D-2HG-treated tumors, αKG supplementation had no additional effect on tumor growth of NCI-H460 cells without IR and displayed tendencies towards increased tumor volume in combination with IR (Fig. 1e). Taken together, our results suggest to a complex metabolic reprogramming induced by CTPI2 treatment compared to octyl-D-2HG treatment.

### Disturbance of cellular and mitochondrial function induced by CTPI2 treatment is enhanced in combination with α-ketoglutarate (αKG) - supplementation in NCI-H460 cells

**Fig. 2:**
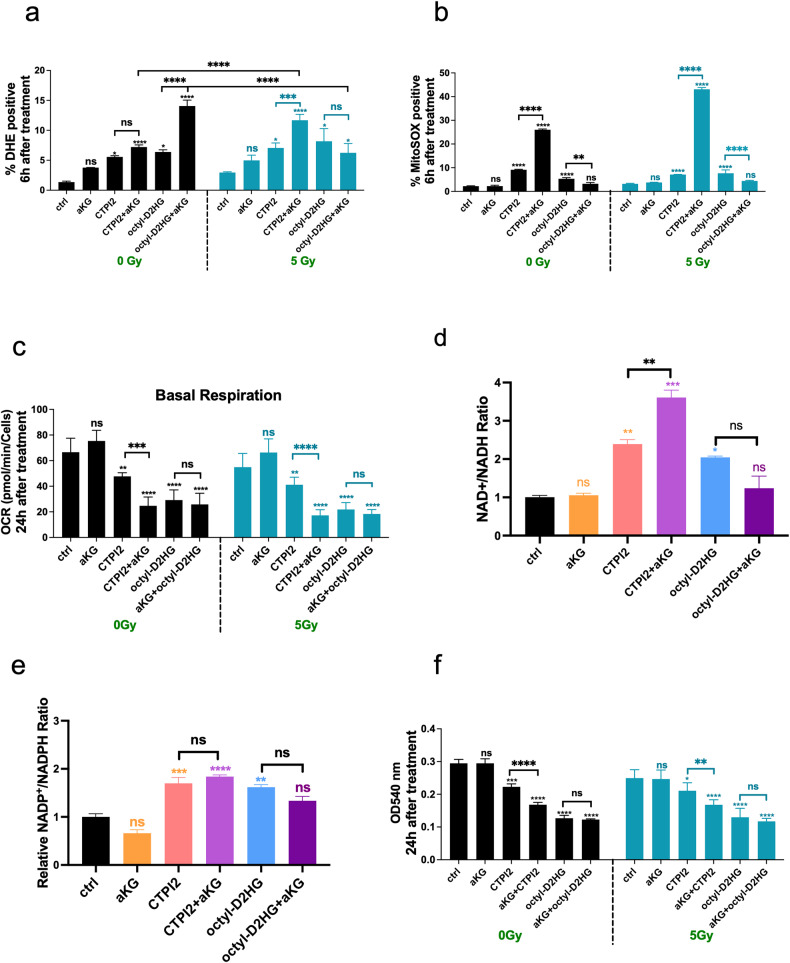
Alteration of cellular and mitochondrial function upon CTPI2 or octyl-D-2HG treatment with or without additional αKG supplementation. Treatments with CTPI2 (200 μM), octyl-D-2HG (150 μM), αKG (8 mM) or the combination of αKG with either of these two treatments were applied to NCI-H460 cells, with or without IR with a dose of 5 Gy. NCI-H460 cells were stained 6 h after indicated treatment with DHE (**a**) or MitoSOX (**b**) to determine cytoplasmic (**a**) or mitochondrial (**b**) ROS by flow cytometry. **c** Basal respiration of mitochondrial function was measured 24 h after indicated treatment by Seahorse XF96 Extracellular Flux analyzer. Relative ratios of NAD^+^/NADH (**d**), NADP^+^/NADPH (**e**) levels normalized to non-treated controls (ctrl) 24 h after respective treatment. **f** Cell proliferation and viability was measured 24 h after treatment by using crystal violet assay. Data represent the mean values (±SD) from three independent experiments (*N* = 3). For statistical analysis one way ANOVA followed by Bonferroni post-test was applied. ns=not significant (*p* > 0.05), *p < 0.05, **p < 0.01, ***p < 0.001, ****p < 0.0001. Asterisks above bars indicate comparison with respective control and parentheses above bars indicate significance between compared groups.

To understand the mechanism behind the effect of αKG supplementation on the DNA damage response and even tumor growth reduction when combined with octyl-D-2HG or CTPI2, short-term effects of respective treatments on the cell function were investigated. Analysis of cytoplasmic reactive oxygen species (ROS) levels by flow cytometry 6 h after the respective treatments alone or in combination with IR at a dose of 5 Gy revealed the highest and significant increase in cytoplasmic ROS levels upon combinatorial treatment of αKG and octyl-D-2HG without IR in NCI-H460 cells (Fig. 2a). A similar trend towards increased cytoplasmic ROS levels was also observed with combinatorial treatment of CTPI2 and αKG, albeit with lower absolute levels of cytoplasmic ROS-positive cells without IR (Fig. 2a). The addition of IR with a single radiation dose of 5 Gy in combination with CTPI2- and αKG-treated NCI-H460 cancer cells increased cytoplasmic ROS levels, whereas no significant effect was observed with the combination of octyl-D-2HG and αKG supplementation (Fig. 2a). Next, the mitochondrial ROS levels were assessed by MitoSOX staining 6 h after treatment. Again, αKG supplementation alone had no significant effect on mitochondrial ROS levels compared to the untreated control group (Fig. 2b). Consistent with cytoplasmic ROS, αKG supplementation strongly potentiated mitochondrial ROS production in NCI-H460 cells pre-treated with CTPI2 (Fig. 2b). In contrast to the potentiating effects on the cytoplasmic ROS production, αKG supplementation in combination with octyl-D-2HG even reduced mitochondrial ROS levels either with or without IR (Fig. 2b). A significant increase in cytoplasmic or mitochondrial ROS levels could lead to the induction of apoptosis and cell death induction [12]. Here, αKG supplementation only potentiated the apoptosis (Fig. S1d) and cell death levels (Fig. S1e) induced by 48 h of CTPI2 treatment in both, irradiated and non-irradiated NCI-H460 cells. No additional effect on apoptosis or cell death levels was observed, when octyl-D-2HG treated NCI-H460 cells were supplemented with αKG (Fig. S1d, e). It was surprising to observe that αKG treatment influenced cell death levels in NCI-H460 cells when combined with IR, whereas αKG treatment without IR was not cytotoxic (Fig. S1e). A comparable increase in cytoplasmic and mitochondrial ROS, and induction of cell death following CTPI2 or octyl-D-2HG treatment alone or in combination with αKG supplementation was confirmed in the A549 lung cancer cell line (Fig. S3c–e).

Since CTPI2 exerts its function on the mitochondrial citrate carrier, the mitochondrial function was measured by using an extracellular flux analyzer (Seahorse Analyzer). Treatment of NCI-H460 cancer cells with αKG alone for 24 h had no significant effect on the basal mitochondrial respiration (Fig. 2c). Again, CTPI2 or octyl-D-2HG treatment alone or in combination with IR, reduced the measured basal respiration (Fig. 2c). Again, αKG supplementation reduced mitochondrial function only in CTPI2-pretreated NCI-H460 cells, whereas αKG- supplementation in combination with octyl-D-2HG had no effect on mitochondrial function (Fig. 2c). Consistent results with each treatment were observed for maximal mitochondrial respiration (Fig. S1f) and mitochondrial ATP production in NCI-H460 cell line (Fig. S1g) and for the basal respiration of A549 cell line (Fig. S3f). Reduction of mitochondrial respiration was associated with the observed increase in ROS production and induction of cell death in NCI-H460 cells [4, 12].

To explain the observed differences in mitochondrial function induced by additional αKG supplementation, the balance of NAD^+^/NADH and NADP^+^/NADPH ratios was examined 24 h after the respective treatments. Additional αKG supplementation in CTPI2-treated NCI-H460 cells significantly increased the ratio of NAD^+^/NADH towards the oxidative state, whereas no significant effect was observed in octyl-D-2HG-treated cells (Fig. 2d). Furthermore, no significant change in the NADP^+^/NADPH ratio was observed upon additional αKG supplementation in either CTPI2- or octyl-D-2HG-treated NCI-H460 cells (Fig. 2e). This was in consistent with the potential of αKG supplementation to enhance mitochondrial ROS production in CTPI2-treated NCI-H460 cells (Fig. 2b). However, αKG supplementation in octyl-D-2HG-treated cells revealed a tendency of the NAD^+^/NADH or NADP^+^/NADPH ratio towards the oxidative state, which was only observed in only octyl-D-2HG-treated NCI-H460 cells (Fig. 2d, e). Remarkably, the applied treatments and their combinations decreased the relative amounts of NAD^+^, NADH, NADP^+^ and NADPH with a higher tendency towards the respective reduced form (Fig. S1h, i). Reduction of redox or energy carrier molecules has previously been linked to effect on cell proliferation [16, 17]. Here, αKG supplementation in combination with CTPI2 further reduced cell viability/proliferation of non-irradiated or irradiated NCI-H460 cells already 24 h after the respective treatments (Fig. 2f). Again, αKG supplementation in the context of octyl-D-2HG treated NCI-H460 cells had no additional effect on reducing cell viability/proliferation in the NCI-H460 cell line (Fig. 2f).

### Nicotinamide (NAM)-supplementation rescued mitochondrial function and reduced DNA damage in CTPI2-treated NCI-H460 cancer cells

As demonstrated in the current study, CTPI2 treatment alone, or in combination with αKG supplementation increased the ratio of NAD^+^/NADH by decreasing the relative amount of NAD^+^ and NADH (Fig. 2d, Fig. S1h). Since nicotinamide (NAM) is the precursor of NAD^+^ [18], we hypothesized that NAM supplementation could shift the ratio of NAD^+^/NADH to the reductive state, restore the decrease in NAD^+^ levels observed with CTPI2 or octyl-D-2HG-treatment, restore the mitochondrial function, and thus rescue radiation-induced DNA damage.

**Fig. 3:**
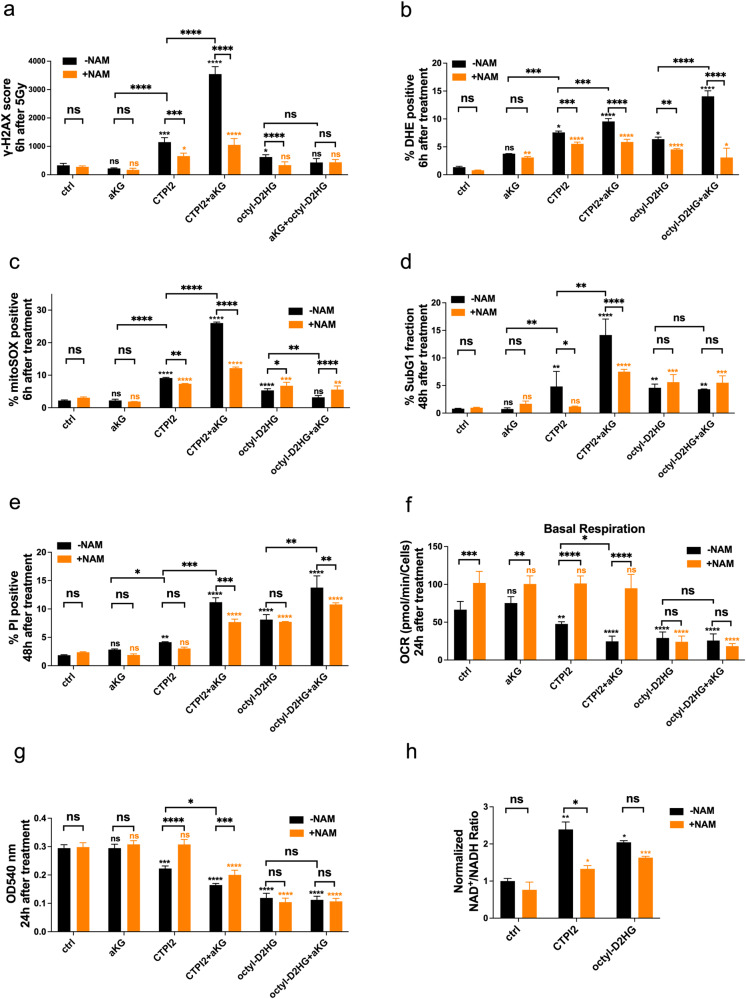
NAM supplementation overcomes mitochondrial dysfunction and reduces DNA damage in CTPI2-treated NCI-H460 cancer cells. NCI-H460 cells were non-treated (ctrl) or pre-treated for 2 h with CTPI2 (200 μM), αKG (8 mM), octyl-D-2HG (150 μM), CTPI2 + αKG, octyl-D-2HG + αKG or additional NAM (1 mM) supplementation as indicated. **a** γ-H2AX signal was assessed by flow cytometry 6 h after the indicated treatment. **b** NCI-H460 cells were stained 6 h after treatment with DHE to determine cytoplasmic ROS by flow cytometry. **c** NCI-H460 cells were stained 6 h after treatment with MitoSOX to determine mitochondrial ROS by flow cytometry. **d** Apoptosis levels were determined 48 h after treatment by analyzing the Sub-G1 fraction by flow cytometry. **e** Cell death levels were investigated by flow cytometry quantifying the % of PI-positive cells 48 h after treatment. **f** Basal respiration of mitochondrial function was measured 24 h after indicated treatment by Seahorse XF96 Extracellular Flux analyzer. **g** Cell proliferation and viability was measured 24 h after treatment using crystal violet assay. **h** Ratios of NAD^+^/NADH levels 24 h after indicated treatments normalized to non-treated controls (ctrl). Black = without NAM (-NAM), Orange = with NAM (+NAM). Data represent the mean values (±SD) from three independent experiments (*N* = 3). One way ANOVA followed by Bonferroni post-test was used to test for statistical significance. ns = not significant (*p* > 0.05), *p < 0.05, **p < 0.01, ***p < 0.001, ****p < 0.0001. Asterisks above bars indicate comparison with respective control and parentheses above bars indicate significance between compared groups.

The assessment of DNA damage by flow cytometric measurement of the γ-H2AX signal or by immunofluorescence-based counting of γ-H2AX foci revealed that supplementation of NAM supplementation was able to overcome the induction of DNA damage in single or combined treatment approaches using CTPI2 or octyl-D2-HG in combination with αKG treatment (Fig. 3a, Fig. S1b). However, NAM supplementation had no effect on the αKG-treated NCI-H460 cell line alone (Fig. 3a). In addition to DNA damage, NAM supplementation also counteracted the effect of CTPI2 or octyl-D-2HG treatment, as well as its combination with αKG, on cytoplasmic ROS levels (Fig. 3b).

In the case of mitochondrial ROS production induced by both CTPI2 and CTPI2 + αKG treatments, NAM supplementation eliminated the mitochondrial ROS levels induced by the respective treatments (Fig. 3c). Unexpectedly, NAM treatment potentiated the mitochondrial ROS production of both octyl-D-2HG and octyl-D-2HG + αKG-treated NCI-H460 cells (Fig. 3c). Consistent with the elimination of mitochondrial ROS, NAM treatment was able to reduce apoptosis levels in CTPI2-treated and in CTPI2 + αKG-treated NCI-H460 cells, whereas no effect was observed in octyl-D-2HG-, octyl-D-2HG + αKG-, αKG- or untreated groups (Fig. 3d). Interestingly, NAM treatment revealed a trend towards reduced cell death levels only in the CTPI2-treated group (*p* = 0.53), but only reached statistically significant differences in cells treated with CTPI2 + αKG or octyl-D-2HG + αKG (Fig. 3e). In addition, we tested potential beneficial effects of NAM- supplementation upon CTPI2 or octyl-D-2HG treatments on the basal mitochondrial function by using extracellular flux analyzer. As depicted in Fig. 3f, NAM treatment for 24 h restored the basal mitochondrial respiration almost to the level of the untreated control group, which was inhibited in CTPI2- or CTPI2 + αKG-treated NCI-H460 cells, but had no effect on octyl-D-2HG-treated NCI-H460 cells alone or in combination with αKG- treatment (Fig. 3f). More important, NAM supplementation demonstrated comparable rescue effect after CTPI2- or CTPI2 + αKG treatment in the A549 cell line on DNA repair (Fig. S3a), cytoplasmic and mitochondrial ROS (Fig. S3c, d), cell death (Fig. S3e), and basal mitochondrial respiration (Fig. S3f).

Furthermore, cell proliferation/viability analysis assessed by crystal violet assay further validated the differences of NAM supplementation in CTPI2- and octyl-D-2HG-treated NCI-H460 cells (Fig. 3g). In our study, reduced cell viability/proliferation induced by CTPI2 or CTPI + αKG treatment was rescued by NAM supplementation (Fig. 3g). However, no significant rescue effect by NAM supplementation was observed in octyl-D-2HG or octyl-D-2HG + αKG-treated groups, highlighting the different changes induced by CTPI2 or octyl-D-2HG treatments (Fig. 3g). To further explore the mechanism behind the rescue effects observed upon NAM supplementation in CTPI2-treated NCI-H460 cells, the relative amount and ratios of NAD^+^/NADH were assessed. Consistent with our previous observations on cellular and mitochondrial function, NAM supplementation only rescued the NAD^+^/NADH ratio in CTPI2-treated NCI-H460 cells, suggesting an increased demand for NAD in CTPI2 treated NCI-H460 cells (Fig. 3h).

### Inhibition of histone-lysin demethylases (KDMs) recapitulated the effects observed with SLC25A1 inhibition by CTPI2

Recent studies have identified D-2HG accumulation as a result of mutations in isocitrate dehydrogenase (IDH), which impairs the function of histone-lysin demethylases 4B (KDM4B), a subgroup of αKG-dependent dioxygenases (αKGDDs), and thereby disrupting local chromatin signaling and suppressing DNA repair by HR [19]. In our recent study, we proposed a strategy to metabolically induce a phenotype mimicking a defect in the HR repair pathway (HRness) by targeting of SLC25A1 and concomitant inhibition of KDM4 through induced accumulation of D-2HG [12]. SLC25A1i, in combination with inhibitors of end-joining (EJ) repair pathways such as PARP, was able to induce context-dependent lethality in vitro and in vivo [12].

**Fig. 4:**
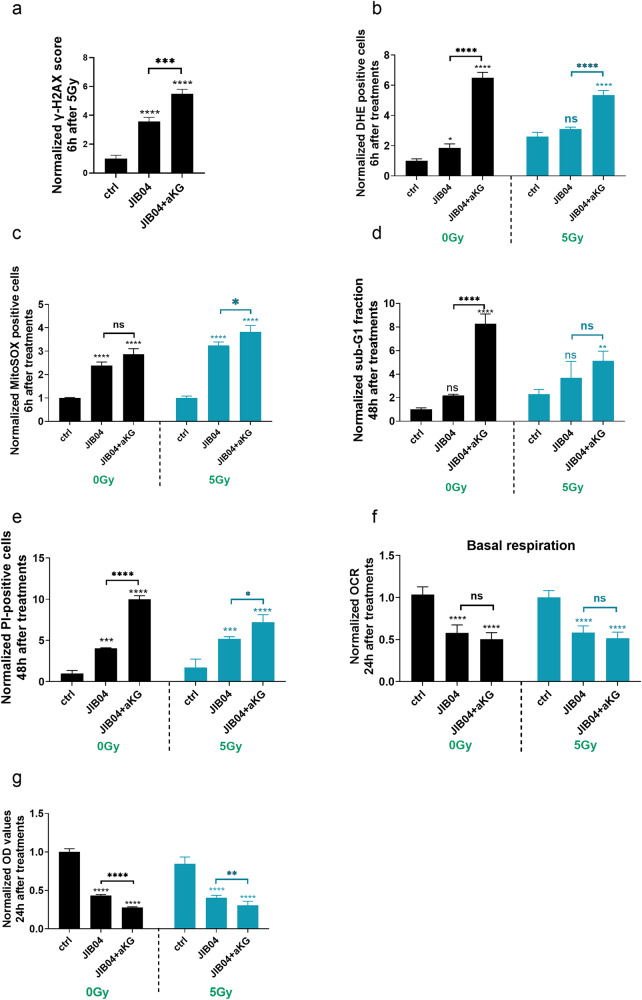
JIB-04 treatment alone or in combination with αKG supplementation recapitulates the effects induced by CTPI2. NCI-H460 cells were exposed to JIB-04 (1 μM), αKG (8 Mm) or the combined treatment of JIB-04 and αKG, with or without IR with a dose of 5 Gy. **a** γ-H2AX signal was measured by flow cytometry 6 h after the indicated treatment in combination with IR (5 Gy). The measured γ-H2AX scores were normalized to non-treated controls (ctrl) to present the increase in γ-H2AX score upon respective treatment. **b** Cytoplasmic ROS were determined 6 h after treatment alone or in combination with IR (5 Gy). Measured %-of DHE positive cells was normalized to the respective untreated control at 0 Gy to present the treatment-induced increase of cytoplasmic ROS. **c** Mitochondrial ROS were determined 6 h after treatment alone or in combination with IR (5 Gy). Measured % of MitoSOX positive cells was normalized to the respective untreated control at 0 Gy to present the treatment-induced increase of mitochondrial ROS. **d** Apoptosis levels (SubG1 fraction) was determined 48 h after indicated treatments upon staining with propidium iodide (PI) diluted in hypotonic buffer by flow cytometry. Measured population of SubG1 positive cells upon indicated treatment was normalized to the non-treated control (ctrl) at 0 Gy. **e** Cell death levels (PI-positive cells) were investigated 48 h after indicated treatments upon staining with propidium iodide (PI) by flow cytometry. Measured population of PI positive cells upon indicated treatment was normalized to the non-treated control (ctrl) at 0 Gy. **f** Basal respiration of mitochondrial function was measured 24 h after indicated treatments by using a Seahorse XFe96 Bioanalyzer. Measured oxygen consumption rate (OCR) values were normalized to the non-treated controls (ctrl). **g** Cell proliferation and viability was measured 24 h after treatment by using the crystal violet assay and the measured OD-values at 540 nm were normalized to non-treated controls (ctrl) as indicated. Data represent the mean values (±SD) from three independent experiments (*N* = 3). One way ANOVA followed by Bonferroni post-test was used for statistical analysis. ns=not significant (*p* > 0.05), *p < 0.05, **p < 0.01, ***p < 0.001, ****p < 0.0001. Asterisks above bars indicate comparison with respective control and parentheses above bars indicate significance between compared groups.

Based on this observation, we wondered if direct KDM inhibition could recapitulate the functional phenotype induced by CTPI2 treatment. To mimic the inhibitory effect of CTPI2 on KDM, NCI-H460 cells were treated with JIB-04, a pan-inhibitor of KDMs. Similar to the effect induced by CTPI2 treatment, JIB-04 treatment stimulated radiation-induced γ-H2AX formation, which was further enhanced by additional αKG supplementation in NCI-H460 (Fig. 4a, Fig. S1c) and A549 cell lines (Fig. S3b). Accordingly, JIB-04-treatment induced cytoplasmic and mitochondrial ROS levels, apoptosis levels and cell death levels of NCI-H460 (Fig. 4b–e) and A549 (Fig. S3c–e) cells without IR. These effects were significantly enhanced by the addition of αKG (Fig. 4b–e). When the cells were treated with IR in addition to JIB-04 treatment alone or in combination with αKG, similar increases in ROS levels and cell death were observed with αKG + JIB-04 treatment (Fig. 4b–e). As an exception, the apoptosis levels were not significantly altered by the described treatments (Fig. 4d). Interestingly, inhibition of JIB-04 for 24 h reduced basal mitochondrial respiration, which was not significantly enhanced by αKG supplementation (Fig. 4f). However, cell viability/proliferation was significantly inhibited after 24 h treatment with JIB-04, and the effect was more pronounced when combined with αKG treatment, whether with or without IR (Fig. 4g). Taken together, inhibition of KDMs was able to recapitulate the effects on DNA repair, mitochondrial and cellular function induced by CTPI2 treatment, suggesting that KDM inhibition is an important factor contributing to the cellular response observed with SLC25A1i.

### α-ketoglutarate (αKG) further radiosensitized NCI-H460 cancer cells treated with CTPI2

**Fig. 5:**
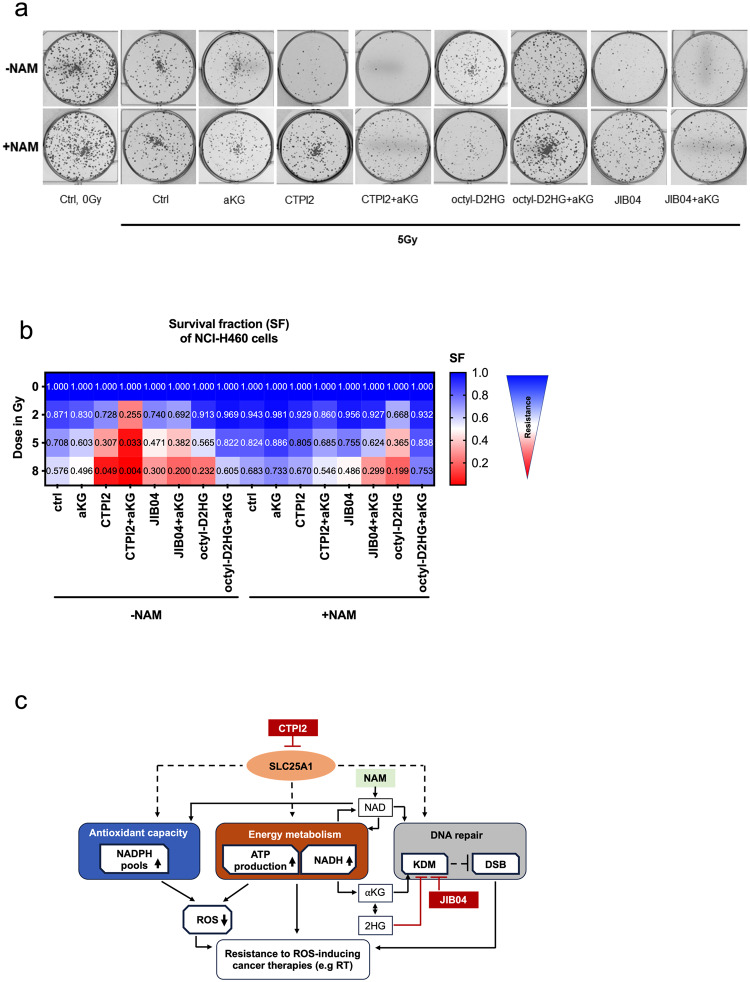
NAM supplementation overcomes treatment-induced radiosensitization in NCI-H460 cells. Colony formation assay was applied to verify the effect of indicated treatments on the long-term survival of NCI-H460 cancer cells upon indicated of IR-doses. NCI-H460 cell line was pre-treated for 2 h with CTPI2 (200 μM), αKG (8 mM), octyl-D-2HG (150 μM), CTPI2 + αKG, octyl-D-2HG + αKG or additional NAM (1 mM) supplementation as indicated, and then irradiated with a dose of 2 Gy, 5 Gy, 8 Gy. Survival fraction (SF) was calculated 8 days after respective treatment. **a** Representative pictures of colony formation after irradiation in combination with indicated treatments. **b** Heatmap representing the mean survival fraction (SF) at different IR-doses (2, 5, 8 Gy) in combination with indicated treatments in NCI-H460 cell line. **c** Schematic representation of SLC25A1-induced metabolic reprogramming. Inhibition of SLC25A1 by CTPI2 impairs cellular antioxidant capacity and energy metabolism leading to accumulation of 2-hydroxyglutarate (2HG), thereby affecting the function of histone lysine demethylases (KDMs) and the repair of radiation-induced double-strand breaks (DSBs). Furthermore, supplementation of α-ketoglutarate (αKG) in combination with CTPI2 potentiated the inhibition of DNA repair, energy metabolism and antioxidant capacity thereby reducing survival after radiotherapy (RT). Direct targeting of KDM by JIB-04 recapitulated the effects of CTPI2, suggesting that KDM inhibition is an important factor contributing to the cellular response observed upon CTPI2 treatment. In addition, nicotinamide (NAM) supplementation rescued the negative effects on DNA repair, antioxidant capacity and energy metabolism observed with CTPI2 treatment, highlighting a potential role for NAD in cellular activities relevant to the survival of irradiated cancer cells upon inhibition of SLC25A1 by CTPI2. ROS reactive oxygen species.

The colony formation assay (CFA) was used to determine the long-term survival of tumor cells after treatment with ionizing radiation [20]. In the present study, the respective treatments were applied in combination with IR to evaluate the long-term radiosensitization effect in the NCI-H460 and A549 cell line. Compared with the irradiated control group, the survival fraction was significantly decreased when the NCI-H460 and A549 cells were treated with CTPI2 in combination with αKG- supplementation (Fig. 5a, b, Figs. S2a–c, S3g, S4). In addition, inhibition of SLC25A1 by CTPI2, of the KDMs by JIB-04 or octyl-D2-HG treatment significantly decreased the survival fraction (SF) of NCI-H460 and A549 cells irradiated at a dose of 5 or 8 Gy (Fig. 5b, Figs. S2b, c, S3g, S4). Again, the reduction in the survival fraction was potentiated by the addition of αKG (Fig. 5b, Figs. S2b, c, S3g, S4). Interestingly, treatment with octyl-D-2HG in combination with αKG supplementation rescued the survival fraction of irradiated NCI-H460 cancer cells compared to octyl-D-2HG treatment alone at irradiation doses of 5 Gy and 8 Gy (Fig. 5b, Fig. S2b, c). It was surprising to observe, that NAM supplementation was able to increase the survival fraction of all indicated treatments, except for the octyl-D-2HG treatment alone or in combination with αKG supplementation (Fig. 5b, Figs. S2b, c, S3g, S4), suggesting an increased cellular requirement for NAD for survival after CTPI2 treatment in combination with IR. However, the pronounced rescue effect of NAM supplementation on the survival of IR-treated NCI-H460 and A549 cells was observed upon CTPI2 + αKG treatment, compared to CTPI2 treatment alone (Figs. 5b, S3g). Our results strongly suggest a global metabolic reprogramming induced by SLC25A1 inhibition alone and in combination with αKG supplementation, resulting in an increased cellular demand for NAD for survival after IR (Fig. 5c). Therefore, the metabolic reprogramming induced by CTPI2 treatment may provide an opportunity for radiosensitization in combination with NAD-producing pathways.

## Discussion

Aerobic glycolysis was reported a century ago by Warburg as a metabolic pathway used in tumor cells, drawing attention to the alterations in cancer metabolism [21, 22]. In the last two decades, the relationship between cancer metabolism and oncogenes has been discovered and further investigated [5, 23]. However, the development of targeted therapies related to metabolic alterations in cancer has been limited in the last decade [24]. The combination of therapies, which could represent one vulnerability for another, has attracted considerable attention in recent years [25, 26]. The development of strategies combining radiotherapy (RT) with drugs targeting phenotype-specific metabolic vulnerabilities to increase the lethality of cancer cells to RT and to overcome the radioresistance associated with metabolic deregulation has been proposed as a new area of research [27, 28].

Our group has reported that genetic (siRNA) or pharmacological targeting of the mitochondrial citrate carrier SLC25A1 by CTPI2 results in accumulation of the oncometabolite D-2HG [11, 12]. Both CTPI2 and cell-permeable D-2HG (octyl-D-2HG) altered the metabolism cancer cell, thereby increasing their sensitivity to RT [12]. As mentioned above, D-2HG is a competitive inhibitor of αKGDD, which uses O_2_ and αKG as cofactors to perform a range of oxidation reactions, such as modification of chromatin or regulation of protein stability [8]. It has been reported that αKG is involved in numerous biological processes, including antioxidant defence, energy production, signaling modules, and genetic modification [29]. In addition, αKG has been used as a dietary supplement and therapeutic agent, i.e. it has been tested and approved for clinical use [29].

To counterbalance the metabolic effects induced by 2-HG accumulation, αKG supplementation was used in our study. Surprisingly, αKG supplementation promoted CTPI2-induced D-2HG production, whereas αKG treatment alone was unable to increase the concentration of D-2HG. The primary source of 2-HG production has been described as the abundance of αKG, which is used as a substrate for the production of 2-HG [30]. However, αKG treatment alone was unable to stimulate the production of D-2HG, suggesting that the reaction conditions didn’t meet the requirements for D-2HG generation. Nevertheless, the combination of CTPI2 treatment in combination with αKG supplementation potentiated the production of D-2HG induced by CTPI2 treatment alone. Our surprising finding suggest that αKG supplementation enhances the necessary conditions for 2-HG production induced by CTPI2 treatment. To further explore the effect of αKG supplementation in combination with CTPI2 treatment on cell biological activities, the combinatorial treatment was tested to investigate their ability to potentiate radiation-induced DNA damage, short-term cell function and cell proliferation, as well as long-term survival.

When radiation-induced DNA damage was examined using the alkaline comet assay, αKG treatment alone was unable to potentiate radiation-induced DNA damage compared to untreated control group. However, αKG supplementation in combination with CTPI2 treatment significantly enhanced radiation-induced DNA damage compared to CTPI2 treatment alone. Similar results were also observed with octyl-D-2HG treatment alone and in combination with αKG supplementation, suggesting that CTPI2-induced DNA damage in combination with ionizing irradiation (IR) is based on the effect of metabolically induced D-2HG production upon CTPI2 treatment. Slightly different results were obtained by monitoring the removal of radiation-induced γ-H2AX signal, as a marker for DNA DSBs [31]. Here, αKG treatment alone stimulated the formation of IR-induced γ-H2AX signal and potentiated the amount of IR-induced γ-H2AX in combination with CTPI2 treatment. On the contrary, αKG treatment in combination with IR did not potentiate the γ-H2AX formation induced by octyl-D-2HG treatment, indicating that CTPI2 and octyl-D-2HG act differently on the repair of IR-induced DSBs [31]. Consistent with the observation on IR-induced DNA damage potentiated by αKG supplementation in combination with CTPI2 treatment, the long-term effect on the survival of IR-treated NCI-H460 and A549 cancer cells measured by colony formation assay revealed that αKG supplementation in combination with CTPI2 treatment potentiated radiosensitization of NCI-H460 and A549 lung cancer cells.

In contrast, αKG supplementation rescued the survival fraction of lung cancer cells treated with octyl-D-2HG. This phenomenon may indicate that the induction of DSBs upon IR, rather than overall DNA damage, correlates with the long-term survival of cancer cells upon IR and thus to radiosensitization as previously described by others [32, 33]. Although αKG treatment enhanced the generation of cytoplasmic and mitochondrial ROS induced by CTPI2 treatment with or without IR, αKG treatment alone had no significant effect on cytoplasmic or mitochondrial ROS generation in NCI-H460 and A549 cancer cells. However, αKG supplementation was able to potentiate the effects of CTPI2 treatment on short-term cell function (mitochondrial respiration, cell death, proliferation) and in vivo tumor growth (CAM assay), whereas αKG supplementation alone had no effect. On the other hand, treatment of NCI-H460 and A549 cancer cells with the oncometabolite octyl-D-2HG did not consistently modulate the measured cellular function compared to CTPI2 treatment. This result underscores the broad multifactorial metabolic reprogramming induced by CTPI2-mediated inhibition of SLC25A1, among which accumulation of D-2HG appears to be an important mechanism affecting cellular function and DNA damage repair upon IR (Fig. 5c). Thus, treatment of cancer cells with octyl-D-2HG, may still allow the cancer cell to exchange citrate between the mitochondria and the cytosol, which seems to be less lethal for the cancer cell and thus less suitable for radiosensitization.

When cell viability/proliferation was analyzed, αKG treatment tended to counteract the inhibition of proliferation induced by octyl-D-2HG treatment alone, suggesting, that αKG supplementation may regain the ability to bind αKG-dependent dioxygenases (αKGDD), thereby restoring their enzymatic function. However, again, αKG supplementation was unable to reverse the inhibition of proliferation induced by CTPI2 treatment, suggesting to distinct effects of octyl-D-2HG application in the presence of functional SLC25A1 and the complex metabolic reprogramming induced by inhibition of SLC25A1 by CTPI2 treatment.

In general, SLC25A1 mediates the transport of citrate between the mitochondria and cytosol, thereby supporting redox homeostasis and lipid metabolism [11, 34–36]. To date, others have reported that accumulation of the 2HG enantiomers L-2HG or D-2HG can occur under certain conditions as pathological metabolites in hypoxic cancer cells produced by lactate dehydrogenase (LDH) or malate dehydrogenase (MDH) [30, 37, 38] or as so-called “oncometabolites” as a result of gain-of-function mutations in the genes encoding for *isocitrate dehydrogenase 1* or *2* (*IDH1 or IDH2*) [39, 40]. We have previously described that inhibition of the citrate export into the cytosol by blocking SLC25A1 using CTPI2 treatment, leads to downregulation of cellular mitochondrial oxidation, accompanied by ROS production and inhibition of DNA repair through the HR pathway by accumulation of D2-HG and accompanied by inhibition of KDMs [12].

Others have linked the metabolic reprogramming of cancer cells to the cellular metabolic phenotype and anabolic state by influencing epigenetic and genetic processes, thereby activating oncogenic cascades [41]. Cellular energy metabolism, mitochondrial function and cellular antioxidant systems are fundamentally regulated/affected by nicotinamide adenine dinucleotide, NAD (including NAD^+^ and NADH) and nicotinamide adenine dinucleotide phosphate, NADP (including NADP^+^ and NADPH) [42]. In particular, the NAD^+^/NADH redox balance not only fuels oxidative phosphorylation (OXPHOS), but also triggers biosynthesis, particularly in the glycolysis pathway and the tricarboxylic acid (TCA) cycle, where NAD^+^ is required as an electron acceptor to maintain glycolysis flux [5]. Elevated NAD^+^ levels enhance glycolysis via glyceraldehyde-3-phosphate dehydrogenase (GAPDH), which requires NAD^+^ as coenzyme [43, 44]. Because of the multifaceted and pathway-connecting role of NAD in the cell, the rate-limiting enzyme for NAD synthesis, nicotinamide phosphoribosyl transferase (NAMPT), has been identified as a target for tumor therapy [45, 46]. NAMPT inhibitors, such as FK866, reduce NAD levels and inhibit cancer cell proliferation by interfering with energy production pathways [46]. Cells with active mitochondrial oxidation require NADH to drive ATP synthesis through the electron transport chain (ETC) [47]. The ETC is the major consumer of NADH, so that dysfunction of the ETC leads to accumulation of mitochondrial and cytosolic NADH [30, 38, 48]. Under these conditions, the activity of the malate dehydrogenase MDH, which is stimulated by αKG accumulation, may help the cells to avoid the accumulation of excess NADH in the cytosol, by MDH-dependent reduction of αKG to 2-HG, which is associated with NADH-oxidation [30, 37, 49]. In our study, αKG supplementation potentiated the accumulation of D2-HG in CTPI2-treated cells, possibly by activating the MDH-dependent reduction of αKG to 2-HG to increase the oxidation of NADH.

Both treatments with CTPI2 or octyl-D2-HG, as well as in combination with αKG- supplementation with CTPI2, shifted the NAD^+^/NADH and NADP^+^/NADPH ratios towards the oxidized form and additionally reduced the amount of NAD^+^, NADH, NADP^+^ and NADPH in the NCI-H460 cancer cells. The high demand for NAD^+^ as an electron acceptor is a common feature of proliferating cancer cells [5]. In an effort to salvage the declining levels of NAD^+^ and other NAD-related species, their precursor nicotinamide (NAM) was added in our study. It was intriguing for us to further investigate whether NAM supplementation would rescue the effect of CTPI2 and octyl-D-2HG on the remodeled biological activities of NCI-H460 and A549 cancer cells. NAM is an amide form of vitamin B3 and the precursor of NAD^+^, an essential co-enzyme of redox reactions for adenosine triphosphate (ATP) production and for several other metabolic processes [50]. In our study NAM supplementation attenuated the level of γ-H2AX signal, thereby reducing the deleterious effect of CTPI2- or CTPI2 + αKG treatments on the formation of radiation-induced DSBs. In addition, NAM supplementation was able to rescue, to varying degrees, the radiosensitization of NCI-H460 and A549 cancer cells induced by treatment with CTPI2, octyl-D-2HG, CTPI2 + αKG or octyl-D-2HG + αKG. These results are consistent with the documented effect of NAM supplementation on genomic stability, which is supported by the function of ATP-dependent DNA repair enzymes [50]. Furthermore, NAM supplementation rescued the effects of CTPI2 alone or in combination with αKG supplementation alleviating induced cytoplasmic/mitochondrial ROS production, apoptosis-, cell death-levels, mitochondrial dysfunction and cell proliferation (Fig. 5c). Our observations suggest that decreasing levels of NAD^+^, NADH, NADP^+^ and NADPH may become critical for cell survival upon inhibition of SLC25A1 in NCI-H460 and A549 cancer cells.

In contrast, no consistent conclusion can be drawn from NAM supplementation experiments in combination with octyl-D-2HG treatment. These observations support the conclusion of different mechanisms induced by octyl-D-2HG treatment compared to CTPI2 treatment. Furthermore, NAM supplementation was used in addition to CTPI2 or octyl-D-2HG to determine the ability of NAM to rescue the oxidative state induced by the latter two treatments. Here, NAM supplementation rescued only the NAD-depletion induced by CTPI2 treatment. NAM supplementation was able to rescue cellular dysfunction in terms of DNA repair, ROS production and induction of cell death induced by CTPI2 treatment. These observations suggest that shifting NAD^+^/NADH ratio back to a more reductive state upon CTPI2 treatment is important for the survival of NCI-H460 and A549 cancer cells upon irradiation.

The salvage pathway, whose rate-limiting enzyme is nicotinamide phosphoribosyl transferase (NAMPT), is the main pathway of NAD^+^ synthesis from NAM, which is the first step of downstream NADH, NADP^+^, NADPH production [50]. A recent study revealed that the inhibition of NAMPT disturbed cell proliferation, mitochondrial function and DNA damage response, which is consistent with the effects induced by CTPI2 in our study [51]. This phenomenon further emphasizes the role of NAMPT in the disturbance of the NAD^+^/NADH ratio induced by CTPI2 and requires further investigation.

A possible involvement of KDM inhibition in the proposed mechanism of action of D-2HG accumulation on DNA repair was investigated by direct inhibition of KDMs using a pan-KDM inhibitor, JIB-04, alone or in combination with αKG supplementation to mimic part of the effects observed with CTPI2 treatment (Fig. 5c). JIB-04 inhibits the demethylase activity of Jumonji enzymes, one of the major KDM subfamilies in the cell, without affecting αKG-dependent prolyl hydroxylases and TET enzymes or other chromatin modifying enzymes such as histone deacetylases [52, 53]. JIB-04 treatment increased radiation-induced γ-H2AX accumulation at 6 h post IR. These results further support the ability of the JIB-04 treatment to interfere with the removal of radiation-induced γ-H2AX and thus potentially interfere with the repair of radiation-induced DNA damage [54].

In addition, the present study provides the first evidence of a combinatorial effect of KDM inhibition by JIB-04 and additional αKG supplementation for radiosensitization, as well as the ability of NAM supplementation to rescue the survival of irradiated NCI-H460 and A549 cancer cells following these treatments. JIB-04 treatment was proposed for use in clinical trials because it altered transcriptional growth programs in cancer cells but not in normal cells, resulting in the induction of cancer-specific cell death induction [53, 54]. Consistent with the reported results, JIB-04 treatment increased the level of cell death and reduced cell proliferation, with or without IR in the present study. Notably, the antineoplastic effect of JIB-04 treatment recapitulated the phenotype induced by CTPI2 treatment and was significantly enhanced by αKG supplementation, consistent with the results obtained with CTPI2 treatment.

In conclusion, the present study describes the synergistic effect of αKG supplementation in combination with SLC25A1 inhibition on cellular and mitochondrial function, creating a cellular demand for NAD to balance cellular activities important for cancer cell survival and radiosensitization. Furthermore, our results provide a new angle for understanding the novel context-dependent role of αKG in the cancer progression and the treatment of cancer.

## Materials and methods

### Cell culture and reagents

The human NSCLC cell lines NCI-H460 and A549 were cultured in DMEM (+d-glucose, +l-glutamine, -pyruvate) media supplemented with 10% FBS and 1% penicillin–streptomycin (Sigma-Aldrich) in a humidified incubator at 37°C and 5% CO_2_. This cell line was obtained from ATCC (Bethesda, MD, USA) and regularly tested for mycoplasma regularly. All chemicals were purchased from Sigma-Aldrich (St. Louis, MO, USA) unless otherwise stated.

### Quantification of D-2HG

The D-2-hydroxyglutarate (D-2HG) Assay Kit (Colorimetric) (BioVision, Milpitas, CA, USA) was used to quantify the intracellular D-2HG levels as previously described [11, 12]. Briefly, 10 million cells were homogenized, lysed and spun down. The supernatant was collected and transferred to a 96-well plate, followed by measurement of the enzymatic conversion of D-2HG to αKG, which could interact with the probe to produce a detectable colored product. Absorbance at 450 nm was measured using a BioTek Synergy H1 microplate reader (BioTek Instruments, Inc., Winooski, VT, USA).

### Irradiation

Irradiation was performed as previously described [2, 11, 55, 56]. Briefly, cells were irradiated at room temperature with an X-ray machine (Precision X-ray Inc., North Branford, CT, USA) operating at 320 kV, 12.5 mA with a 1.65 mm Al filter, at a distance of 50 cm and a dose rate of 3.71 Gy/min. Cells were returned to the incubator immediately after exposure to ionizing radiation (IR).

### Alkaline comet assay

To quantify the DNA damage levels, the alkaline comet assay was performed as previously described [12]. Cells were plated in in triplicate in 12 well plates at a cell density of 200,000 cells per well. Treatments were administered at different concentrations 24 h after plating. Slides were covered with 1% low melting point (LMP) agarose to form the first layer for gel retention. The second or cell-containing layer was a mixture of 30% cell-containing medium and 70% 1% LMP agarose. Slides were placed in a lysis solution (containing1.2 M NaCl, 100 mM Na_2_EDTA, 0.1% sodium lauryl sarcosinate and 0.26 M NaOH, pH > 13) for 1 h at 4 °C after the agarose gel had solidified. The slides were then placed in freshly prepared alkaline electrophoresis solution (containing 2 mM Na_2_EDTA and 0.03 M NaOH, pH = 12.3) for 10 min before electrophoresis. Electrophoresis was performed at 20 V for 1 h. The slides were then immersed in water and 100% ethanol to remove excess electrophoresis solution. Propidium iodide (PI) was used to detect the DNA under fluorescence microscopy.

### Colony formation assay

The effect of different treatments on long-term survival was analyzed by clonogenic survival analysis as described previously [2, 12]. Different densities (200, 400, 800, 1600, 3200 cells per well) were plated in the 6-well plates and treated with the indicated drug concentrations 24 h later. Radiation was initiated 2 h after drug treatment at 2 Gy, 5 Gy, 8 Gy separately. Non-irradiated cells were sham irradiated at room temperature for the same period of time as their irradiated counterparts. After 8–10 days, colonies were stained with methanol containing 0.1% (w/v) Coomassie Blue dye and counted manually.

### Flow cytometry analysis

30,000 cells were plated in 6-well plates 24 hours before treatment. The cell supernatant was collected in flow cytometry tubes before cells were trypsinized with Accutase (PAN Biotech, Germany). The detached cells were equally aliquoted into 3 flow cytometry tubes before centrifugation (1500 rpm, 5 min) and the supernatant was discarded. Cells were then stained separately for different purposes with the following staining solutions: (a) Cytoplasmic ROS levels: 0.5 nM DHE diluted in PBS; (b) Mitochondrial ROS levels: 5 μM MitoSOX staining solution (Invitrogen, USA) diluted in DMEM (+d-glucose, +l-glutamine, −pyruvate) medium; (c) Apoptosis: 5 µg/ml PI diluted in hypotonic buffer (0.05% Triton X-100 + 0.1% sodium citrate in PBS); (d) Cell death: 1 µg/ml PI diluted in PBS.

(e) To quantify DNA damage, we recorded the time-dependent formation and resolution of γH2A.X foci using flow cytometry, cells were fixed with Fix-Perm solution (Bioscience™ Foxp3/Transcription Factor Staining Buffer Set, Invitrogen, USA) for 1 h before staining with γH2AX staining solution for 0. 5 h. The γH2AX staining solution consisted of γH2AX antibody (γH2AX (Alexa Fluor 647), BD Pharmingen, USA, #AB_1645414) at a ratio of 1:100 in permeable buffer (Bioscience™ Foxp3/Transcription Factor Staining Buffer Set, Invitrogen, USA). After staining, cells were transferred to flow cytometry tubes for measurement. Finally, the γH2AX level was calculated as follows:

#### γH2AX score = % γH2AX positive cells * fluorescence intensity of γH2AX positive cells

Here, γ-H2AX foci readings obtained by flow cytometry were confirmed by γ-H2AX foci readings obtained by fluorescence microscopy, as described, before the γ-H2AX antibody was diluted 1:100 in permeable buffer before staining the cells, as previously described [12]. CytoFLEX flow cytometer (Beckman Coulter, Inc. USA) was used to analyze the samples.

### γH2AX foci detection by immunofluorescence

Cells were fixed with fixation/permeabilization solution (4% PFA and 0.2% Triton X-100 in PBS) for 15 min at room temperature after treatment and then blocked with blocking solution (2% normal goat serum (NGS) in PBS) for 30 min at room temperature. For γH2AX staining, cells were stained with γH2AX antibody (Alexa Fluor® 647 mouse anti-H2AX (pS139), BD Biosciences, USA, #AB_1645414) coupled to Alexa Fluor 647 at 1:50 dilution in blocking solution for 1 h at room temperature. For Rad51 staining, 1:100 rabbit Rad51 (Anti-Rad51 (Ab-1) Rabbit pAb, Millipore, USA, #PC130) was used in blocking solution and incubated for 30 min on a shaker at 4 °C. DNA was stained with Hoechst33342 (3 µM in PBS) for 30 min at RT. Coverslips were mounted on glass slides with DAKO mounting medium (Dako NA Inc., Carpinteria, CA, USA). Nuclear foci were manually scored using the AxioObserver.Z1 fluorescence microscope (with Apotome) (Zeiss, Oberkochen, Germany). γH2AX foci in at least 50 cells per slide were counted.

### Crystal violet assay

To quantify the change of cell proliferation/viability induced by the treatments, the crystal violet assay was used as previously described [12]. Briefly 5000 cells per well were seeded in a 96-well plate and incubated at 37 °C for 24 h prior to treatment. The medium was discarded before fixation with 1% glutaraldehyde, followed by the addition of 0.1% crystal violet staining solution. 0.2% Triton-X 100 was used to lyse the cells. Finally, the absorbance at the wavelength of 540 nm (OD540) was measured by using the BioTek Synergy H1 microplate reader (BioTek Instruments, Inc., Winooski, VT, USA).

### Cell redox state determination

NAD^+^, NADP^+^, NADH and NADPH levels, and NAD^+^/NADH and NADP^+^/NADPH ratios were determined using the NAD/NADH-Glo™ and NADP/NADPH-Glo™ Assays kits (Promega, USA) according to the manufacturer’s protocol. Briefly, 10,000 cells were plated in a 96-well plate 24 h prior to treatment. The assay procedure started with cell lysis followed by analysis of NAD(P)^+^ or NAD(P)H separately. The ratio of NAD^+^/NADH and NADP^+^/NADPH was calculated according to the instruction of the kits, as previously described [12].

### Mitochondrial function analysis (Seahorse technology)

Following the previously described steps, we seeded 10,000–15,000 cells were seeded into each well, excluding the four corners for background correction, of a Seahorse XF 96-well plate and the plate was incubated overnight at 37 °C in 5% CO_2_. The cell culture medium was replaced with 180 μl of Seahorse XF DMEM media (Seahorse XF DMEM Media (with HEPES) + 1 mM pyruvate, 2 mM glutamine, 10 mM glucose) and the cells were incubated for 45 min at 37 °C in a CO_2_-free incubator before measurement. Oxygen consumption rate (OCR) and extracellular acidification rate (ECAR) were measured using a Seahorse XF96 Analyzer (Agilent, Santa Clara, USA). OCR was determined in four consecutive steps: (1) no treatment, (2) oligomycin (1 μM), (3) carbonyl cyanide-4-(trifluoromethoxy) phenylhydrazone (FCCP, 2 μM), (4) rotenone and antimycin A (0.5 μM). For cell number normalization of individual wells, DNA content fluorescence was measured after cells were stained with 10 μg/mL Hoechst 33342 solution (Sigma-Aldrich) solution after each assay. Data were analyzed using Wave 2.6.1 software (Agilent Technologies). All metabolic parameters were normalized to Hoechst intensity (relative fluorescence units, RFU) in each well as previously described [4, 11, 12, 55].

### Tumor growth assessment using an in vivo CAM-model

The chick embryo chorioallantoic membrane (CAM) assay was used as an in vivo model to study the effect of indicated treatments on the tumor growth of NCI-H460 cells. Chicken eggs were incubated in the environment of relative air humidity of 65% and a temperature of 37 °C, with automatic turning 4 times a day for 10 days before grafting to ensure proper embryo development. On the day of grafting, large vessel area was marked by candling the eggshell before opening a “window”. A hole was made in the bottom of the eggs with scissors and widened with tweezers to allow the CAM to be lowered. The selected window was opened with a drill. Two million cells were dissolved in 50 μL PBS and pipetted onto the CAM of the chicken eggs and the window was sealed with tape. Seven days after grafting, the tumors were dissected and their diameters measured as previously described [12, 13, 57].

### Statistical evaluation

Statistical analysis was performed by using GraphPad Prism 7.0. Calculations of various formulas were performed by using Microsoft Excel 2019. Experiments were repeated 3 times. Assuming a normal distribution, statistical significance was calculated. Either unpaired Student´s t-test or the two-way analysis of variance (ANOVA) with Bonferroni post-hoc test was used. The confidence interval was set at 95%. The significance level was set at *α* = 0.05 (equivalent to 5%), i.e., the difference between two data sets was considered significant if the p-value was ≤0.05. Significances are indicated by asterisks (*) in the figures. Here, *p < 0.05 stands for significant, **p < 0.01 for highly significant, ***p < 0.001 for extremely significant and ****p < 0.0001 for most significant, ns not significant.

### Supplementary information

Supplementary Material

## Data Availability

Any additional information required to reanalyze the data reported in this paper is available from the lead contact upon request.

## Code availability

This study did not use any unpublished custom code, software, or algorithm.
