# Supplementary material for: α-Ketoglutarate supplementation and NAD+ modulation enhance metabolic rewiring and radiosensitization in SLC25A1 inhibited cancer cells
Source: Cell Death Discov. 2024 Jan 15;10:27. doi: 10.1038/s41420-024-01805-x (PMC10789775; doi:10.1038/s41420-024-01805-x)
Supplement: Supplementary file 1 — Supplementary Material [file 41420_2024_1805_MOESM1_ESM.docx]

Supplementary Material

**α-Ketoglutarate Supplementation and NAD+ Modulation Enhance Metabolic Rewiring and Radiosensitization in SLC25A1 Inhibited Cancer Cells**

Kexu Xiang^1,2,3^, Mikhail Kunin^1^, Safa Larafa^1^, Maike Busch^4^, Nicole Dünker^4^, Verena Jendrossek^1,5^, Johann Matschke^1,5*^

^1^Institute of Cell Biology (Cancer Research), University Hospital Essen, University of Duisburg-Essen, 45147 Essen, Germany

^2^ Department of Gastroenterology, Chongqing University Cancer Hospital, 400030 Chongqing, China.

^3^Chongqing Key Laboratory of Translational Research for Cancer Metastasis and Individualized Treatment, Chongqing University Cancer Hospital, 400030 Chongqing, China.

^4^ Center for Translational Neuro- and Behavioral Sciences, Institute of Anatomy II, Department of Neuroanatomy, Medical Faculty, University of Duisburg-Essen, 45147 Essen, Germany.

^5^ German Cancer Consortium (DKTK), partner site Essen, a partnership between DKFZ and University Hospital Essen, Germany.

* Correspondence: [Johann.Matschke@uk-essen.de](mailto:Johann.Matschke@uk-essen.de) (J.M.), Institute of Cell Biology (Cancer Research), University Hospital Essen, University of Duisburg-Essen, Virchowstrasse 173, 45147 Essen, Germany; Phone: +49-201-7234234; Fax: +49-201-7235904; E-mail: johann.matschke@uk-essen.de.


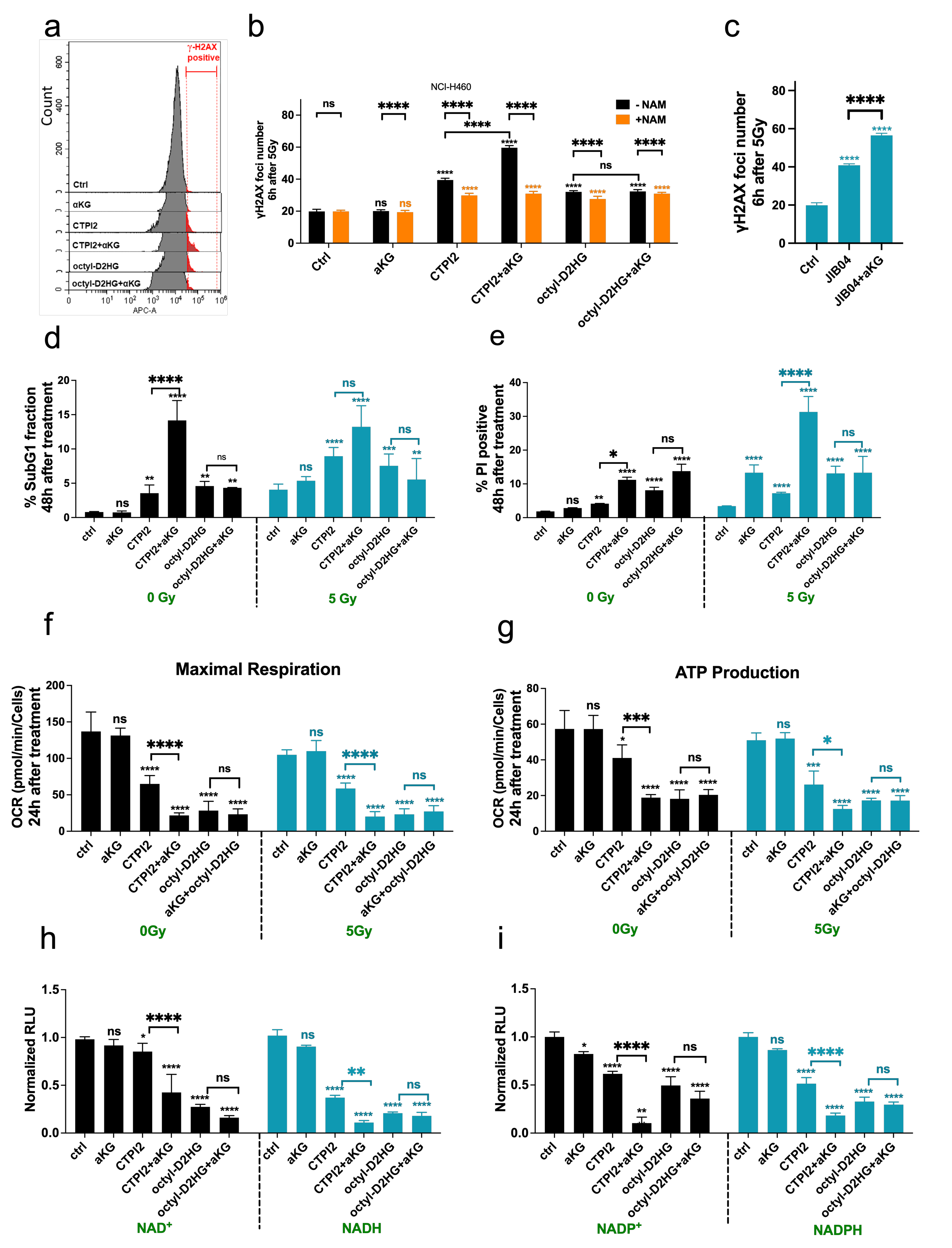


**Figure S1: Alterations of cellular and mitochondrial function and DNA repair induced by CTPI2 or octyl-D-2HG treatment with or without additional αKG supplementation.**

NCI-H460 cells were pre-treated for 2h with CTPI2 (200μM), αKG (8mM), octyl-D-2HG (150μM), CTPI2+αKG or octyl-D-2HG+αKG, and then irradiated with a dose of 0Gy or 5Gy. **(a)** Representative flow cytometry histogram depicting measured γ-H2AX signal 6h after indicated treatment in NCI-H460 cells. **(b)** γ-H2AX foci number was counted 6 h after drug treatment with IR (5Gy) as indicated in NCI-H460 cell line without NAM (-NAM) or with NAM (+NAM, 1mM). **(c)** γ-H2AX foci number was counted 6 h after JIB04 (1µM) +/- αKG treatment with IR (5Gy) as indicated in NCI-H460 cell line. γH2AX foci in at least 50 cells per slide were counted. **d, e)** Apoptosis (%Sub-G1 population) **(d)** and cell death (%PI-positive cells) **(e)** levels were determined by flow cytometry 48h after treatments as indicated. **f, g)** Mitochondrial function was measured 24h after indicated treatments by Seahorse XF96 Extracellular Flux analyser with the utilization of mitochondrial stress test. Mitochondrial function, including mitochondrial maximal respiration **(f)** and mitochondrial ATP production **(g)**, were measured 24h after CTPI2 or octyl-D-2HG treatment, with or without IR. **h, i)** Relative amounts of NAD^+^, NADH **(h)**, NADP^+^ and NADPH **(i)** levels as measured by relative luminescence units (RLU) in NCI-H460 cells 24h after respective treatment. Data represent the mean values (±SD) from three independent experiments (N=3). For statistical analysis one way ANOVA followed by Bonferroni post-test was applied. ns=not significant (p > 0.05), * p < 0.05, ** p < 0.01, *** p < 0.001, **** p < 0.0001. Asterisks above bars indicate comparison with respective control and parentheses above bars indicate significance between compared groups.


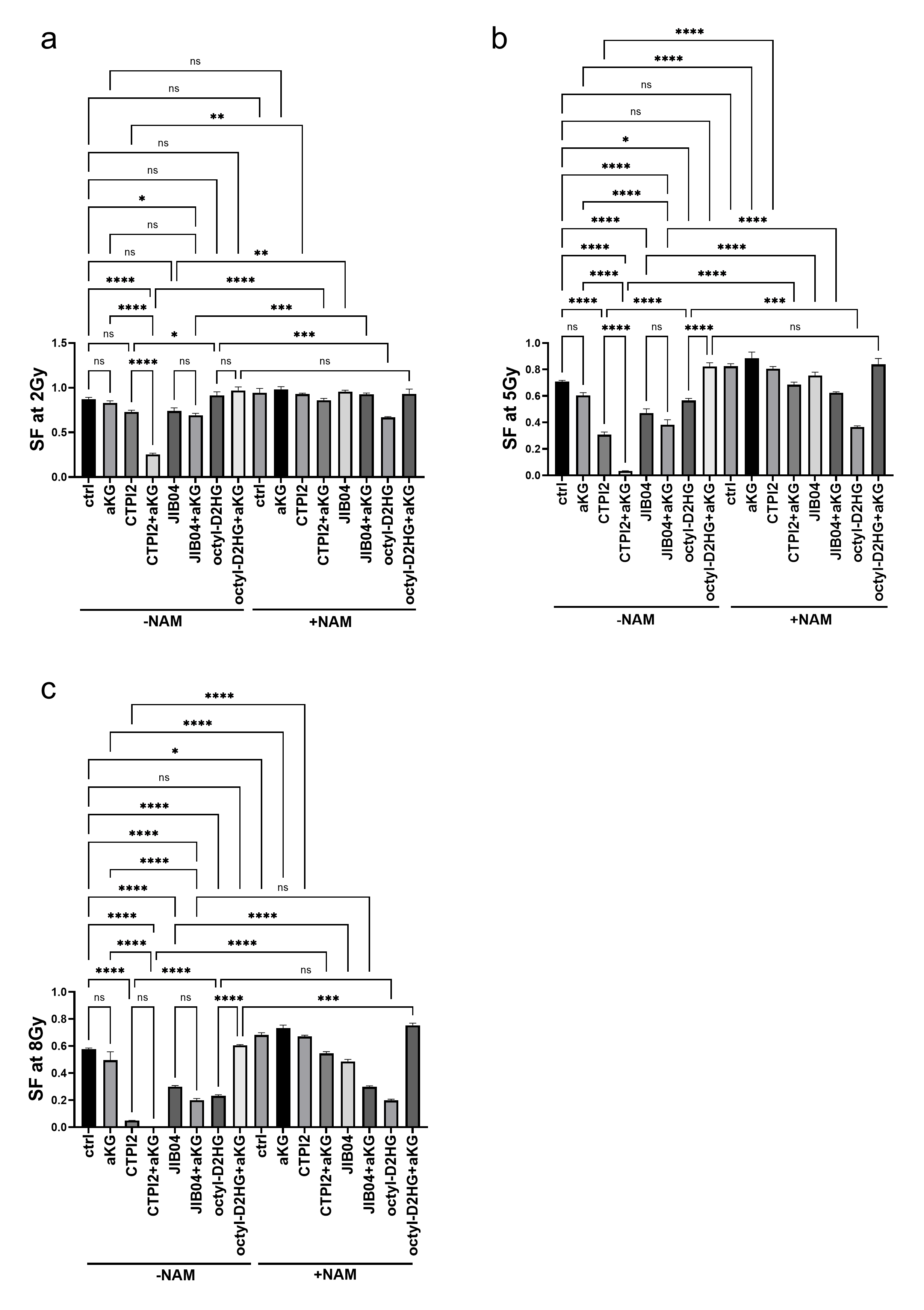


**Figure S2: Survival fraction of NCI-H460 cells upon indicated treatments in combination with IR.**

NCI-H460 cell line was pre-treated for 2h with CTPI2 (200μM), αKG (8mM), octyl-D-2HG (150μM), CTPI2+αKG, octyl-D-2HG+αKG, JIB-04 (1μM), JIB-04 (1μM)+αKG or additionally NAM (1mM) supplementation as indicated, and then irradiated with a dose of 2Gy **(a)**, 5Gy **(b)**, 8Gy **(c)** separately. Survival fraction (SF) was calculated 8 days after respective treatment. Data represent the mean values (±SD) from three independent experiments (N=3). One way ANOVA followed by Bonferroni post-test was applied. * p < 0.05, ** p < 0.01, *** p < 0.001, **** p < 0.0001. Asterisks above bars indicate comparison with respective control and parentheses above bars indicate significance between compared groups.

**
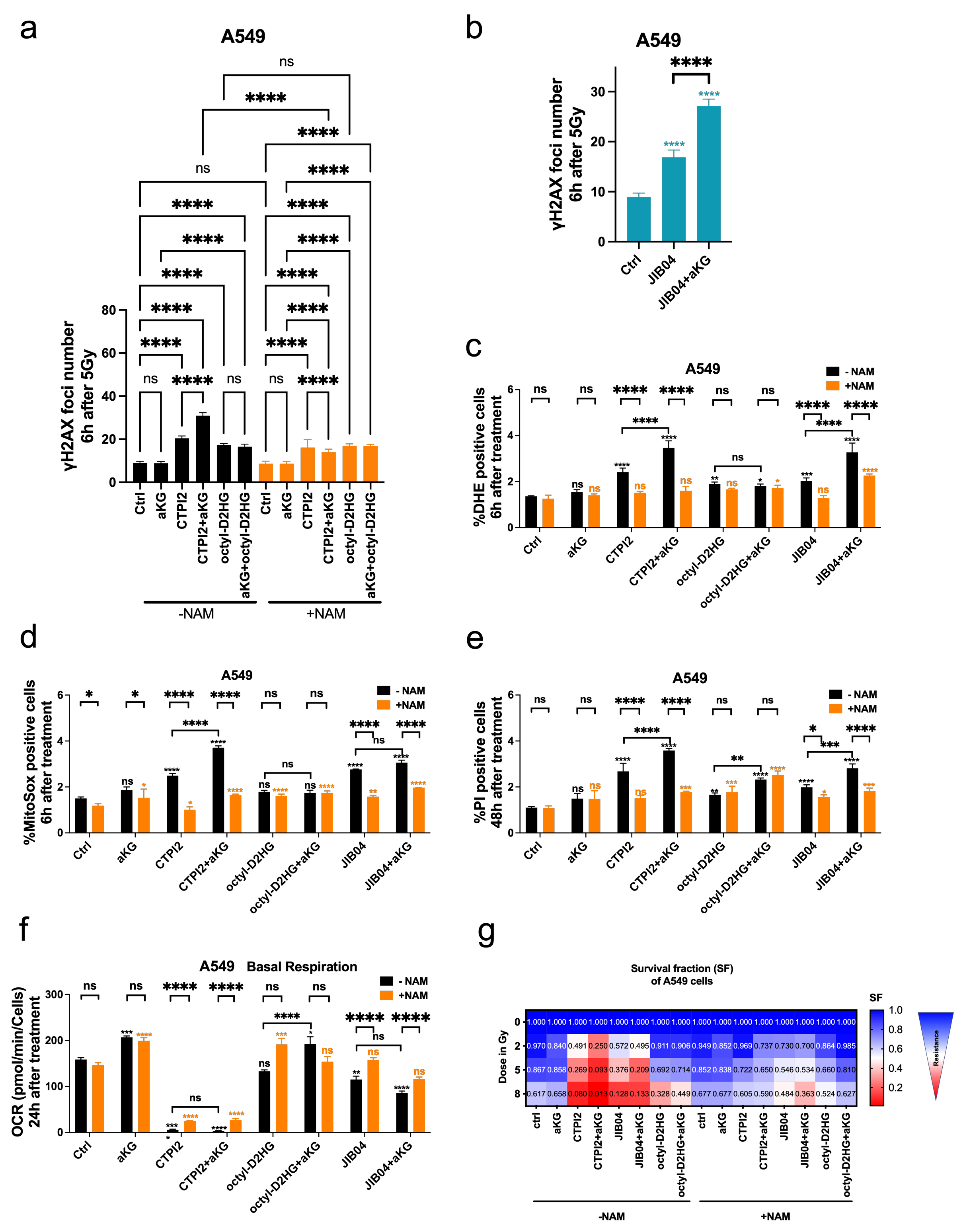
**

**Figure S3: Treatments with CTPI2 or Octyl-D-2HG alter cellular and mitochondrial function and DNA repair in A549 cell line, with or without additional αKG supplementation.**

A549 cell line was pre-treated for 2h with CTPI2 (200μM), αKG (8mM), octyl-D-2HG (150μM), CTPI2+αKG, octyl-D-2HG+αKG, JIB-04 (1μM), JIB-04 (1μM)+αKG or additionally NAM (1mM) supplementation as indicated, and then irradiated with a dose of 5Gy **(a,b)** or 2-8Gy for (**g**). **(a)** γ-H2AX foci number was counted 6h after drug treatment with IR (5Gy) as indicated in A549 cell line without NAM (-NAM) or with NAM (+NAM, 1mM). **(b)** γ-H2AX foci number was counted 6h after JIB04 (1µM) +/- αKG treatment with IR (5Gy) as indicated in A549 cell line. γH2AX foci in at least 50 cells per slide were counted. **c)** A549 cells were stained 6h after treatment with DHE **(c)** or MitoSOX **(d)** to determine cytoplasmic **(c)** or mitochondrial **(c)** ROS by flow cytometry. **e)** Cell death levels of A549 cell line were investigated by flow cytometry quantifying the % of PI-positive cells 48h after treatment. **f)** Basal mitochondrial respiration (Oxygen consumption rate, OCR) of A549 cell line was measured 24h after indicated treatment by Seahorse XF96 Extracellular Flux analyser. **g)** Heatmap representing the mean survival fraction (SF) at different IR-doses (2, 5, 8Gy) in combination with indicated treatments in A549 cell line. Colony formation assay was applied to verify the effect of indicated treatments on the long-term survival of A549 cancer cells upon indicated of IR-doses. A549 cell line was pretreated for 2h with CTPI2 (200μM), αKG (8mM), octyl-D-2HG (150μM), CTPI2+αKG, octyl-D-2HG+αKG, JIB04, JIB04+ αKG or additional NAM (1mM) supplementation as indicated, and then irradiated with a dose of 2Gy, 5Gy, 8Gy. Survival fraction (SF) was calculated 8 days after respective treatment. Data represent the mean values (±SD) from three independent experiments (N=3). One way ANOVA followed by Bonferroni post-test was applied. * p < 0.05, ** p < 0.01, *** p < 0.001, **** p < 0.0001. Asterisks above bars indicate comparison with respective control and parentheses above bars indicate significance between compared groups.


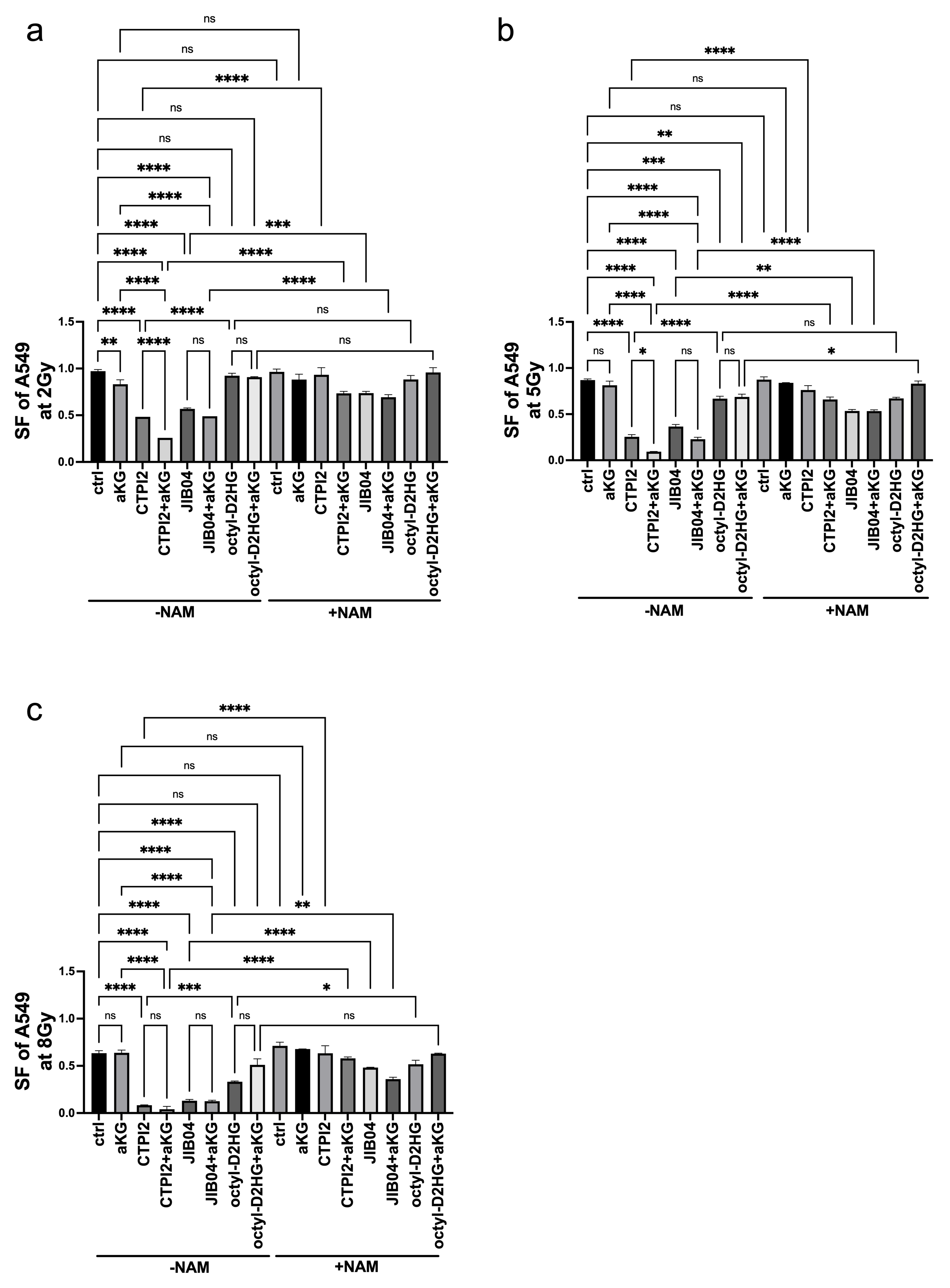


**Figure S4: Survival fraction of A549 cells upon indicated treatments and indicated irradiation doses.**

Colony formation assay was applied to verify the effect of indicated treatments on the long-term survival of A549 cancer cells upon indicated of IR-doses. A549 cell line was pretreated for 2h with CTPI2 (200μM), αKG (8mM), octyl-D-2HG (150μM), CTPI2+αKG, octyl-D-2HG+αKG, JIB04, JIB04+ αKG or additional NAM (1mM) supplementation as indicated, and then irradiated with a dose of 2Gy, 5Gy, 8Gy. Survival fraction (SF) was calculated 8 days after respective treatment. **a-c)** Survival fraction (SF) of A549 cell line represented as a bar chart under respective treatments as indicated per radiation dose **(a)** 2Gy, **(b)** 5Gy, **(c)** 8Gy. Data represent the mean values (±SD) from three independent experiments (N=3). One way ANOVA followed by Bonferroni post-test was applied. * p < 0.05, ** p < 0.01, *** p < 0.001, **** p < 0.0001. Asterisks above bars indicate comparison with respective control and parentheses above bars indicate significance between compared groups.
